# Impact of Semaglutide on fat mass, lean mass and muscle function in patients with obesity: The SEMALEAN study

**DOI:** 10.1111/dom.70141

**Published:** 2025-10-09

**Authors:** Mathieu Alissou, Thomas Demangeat, Vanessa Folope, Hélène Van Elslande, Hélène Lelandais, Julia Blanchemaison, Pierre‐Emmanuel Cailleaux, Suzan Guney, Alexandra Aupetit, Agnès Aubourg, Clément Rapp, André Petit, Morgane Godin, Luc Vignal, Sébastien Grigioni, Pierre Déchelotte, Guillaume Colange, Moïse Coëffier, Najate Achamrah

**Affiliations:** ^1^ Department of Nutrition and CIC‐CRB 1404, CSO Rouen Normandie CHU Rouen Rouen France; ^2^ Univ Rouen Normandie, INSERM, Normandie Univ ADEN UMR 1073 “Nutrition, Inflammation and Microbiota‐Gut‐Brain Axis” Rouen France

**Keywords:** antiobesity drug, body composition, obesity care, Semaglutide

## Abstract

**Aims:**

Semaglutide, a GLP‐1 receptor agonist, has shown efficacy in promoting weight loss. However, limited data exist on its impact on lean mass, muscle function, and metabolic adaptations. The SEMALEAN study aims to evaluate these parameters in patients with obesity treated with Semaglutide (2.4 mg).

**Materials and Methods:**

This prospective study enrolled 115 patients with obesity between February 2022 and November 2024. Body weight, body composition (measured by DXA), muscle function (handgrip strength), and resting energy expenditure (REE) were assessed at baseline (M0), 7 months (M7), and 12 months (M12). Subgroup analyses examined the impact of sex, type 2 diabetes, previous GLP‐1 use, and history of bariatric surgery.

**Results:**

A total of 106 patients (68.9% female; mean BMI 46.3 kg/m^2^) completed the study. Weight loss was significant, with mean reductions of 10% at M7 and 13% at M12; 59% of patients achieved ≥10% weight loss. Total fat mass decreased by 14% at M7 and 18% at M12, while lean mass initially declined (−3 kg at M7) but stabilised thereafter. Handgrip strength improved significantly (+4.5 kg at M12), and the prevalence of sarcopenic obesity decreased from 49% at baseline to 33% at M12. REE normalised to lean mass increased significantly from M7 to M12. Subgroup analyses revealed greater weight and fat mass loss in women, while patients with type 2 diabetes or prior GLP‐1 analogue use showed attenuated responses. Patients with a history of bariatric surgery exhibited the most pronounced reductions in body composition parameters.

**Conclusions:**

The SEMALEAN study highlights the significant impact of Semaglutide 2.4 mg on weight loss, fat mass reduction, and muscle function improvement, with preserved lean mass and metabolic efficiency. These findings underscore the importance of a comprehensive approach to obesity management, addressing not only weight loss but also functional and metabolic adaptation.

## 1 INTRODUCTION

Obesity has become a significant public health challenge due to its rising prevalence^1^ and associated comorbidities including: type 2 diabetes, cardiovascular disease, obstructive sleep apnea, osteoarthritis, cancers (breast, colorectal…).^2^ In addition to lifestyle interventions and bariatric surgery, pharmacological treatments have emerged to improve weight management outcomes.^3^ Among these, glucagon‐like peptide‐1 (GLP‐1) receptor agonists have shown substantial efficacy in inducing weight loss. GLP‐1 is an incretin hormone released from enteroendocrine cells in the gut in response to food intake.^4^ Initially developed to manage hyperglycemia in type 2 diabetes, GLP‐1 receptor agonists, such as Semaglutide, are now recognised for their potent effects on appetite regulation, gastric emptying, and food intake. Semaglutide, administered once weekly at a dose of 2.4 mg, has demonstrated significant weight loss outcomes in clinical trials, with reductions in body weight exceeding 15% in many patients.^5^, ^6^ These outcomes highlight its potential as a therapeutic option for obesity, particularly in cases where lifestyle interventions alone are insufficient.

Despite the efficacy of GLP‐1 receptor agonists in reducing body weight, limited data exist on their impact on body composition, including changes in fat mass, lean mass, muscle function, and the potential development of sarcopenia defined by a loss of muscle mass and function.^7^ Current data suggest a preservation of muscle mass and function during Semaglutide treatment. However, these data are highly heterogeneous. Few studies have used DXA,^6^, ^8^ the gold standard for measuring body composition, and even fewer have assessed muscle function.^9^, ^10^ Data are very limited for the highest dose of Semaglutide (2.4 mg), as most studies have focused on the 1 mg dose.^8^, ^9^, ^10^, ^11^

Preserving muscle mass during weight loss is critical, as sarcopenia is associated with an increased risk of morbidity and mortality,^12^ particularly in patients with obesity.^13^ Muscle mass is also a key determinant of energy expenditure. Low energy expenditure and impaired fat oxidation are risk factors for weight gain, but also cause resistance to weight loss.^14^ Thus, assessing the effects of Semaglutide on lean mass and muscle function is critical to fully evaluating the benefits and risks of such treatments, and to focus more on the quality of weight loss and its maintenance rather than the absolute weight loss.

The SEMALEAN study aims to investigate the effects of Semaglutide on body composition, focusing on lean mass and muscle function over 1 year of treatment.

## 2 MATERIALS AND METHODS

The SEMALEAN study is a prospective longitudinal trial conducted at the Nutrition Department of Rouen University Hospital, Normandy. The study was conducted in accordance with the principles of the Declaration of Helsinki and Good Clinical Practice guidelines. The protocol was approved by an independent ethics committee.

### 2.1 Study population

The SEMALEAN study was conducted as a prospective, longitudinal, real‐world cohort within the framework of the French temporary use authorization (ATU) and early access programme for Semaglutide (Wegovy®). All consecutive adult patients initiating semaglutide treatment at our centre between June 2021 and November 2024 were screened. Eligible patients met the inclusion criteria of grade 3 obesity (BMI ≥40 kg/m^2^) with at least one obesity‐related comorbidity (such as obstructive sleep apnea syndrome, hypertension, dyslipidemia, or cardiovascular disease) and documented failure of lifestyle interventions (including dietary changes and increased physical activity). Patients were excluded if they met any of the following criteria: active cancer or a history of pancreatitis, premature discontinuation of treatment before the seventh month, or incomplete follow‐up data.

No retrospective patient selection was performed.

The study was conducted in compliance with the principles of the Declaration of Helsinki.

### 2.2 Study design

All enrolled patients were evaluated at three key time points: baseline (M0)—before the initiation of Semaglutide; midpoint (M7)—at 7 months of treatment; completion (M12)—at 12 months of treatment. Semaglutide treatment began with a weekly dose of 0.25 mg, which was progressively increased every 4 weeks through the following doses: 0.5 mg, 1 mg, 1.7 mg, and reaching the target dose of 2.4 mg by the 16th week.

Exploratory subgroup analyses were also conducted according to prior exposure to GLP‐1 receptor agonists, given the increasing number of patients transitioning between GLP‐1 therapies in clinical practice. Patients previously exposed to Semaglutide before the study were assigned to the next dose escalation level.

### 2.3 Data collection and measurements

Clinical and demographic data were collected during the initial visit. Follow‐up visits involved an evaluation of body weight, waist circumference, body composition, energy expenditure, and muscle function:

#### 2.3.1 Weight and waist circumference

Weight changes were recorded both as absolute values (kilograms) and percentages relative to baseline.

Waist circumference was measured (centimetre) at the midpoint between the lower margin of the last palpable rib and the top of the iliac crest, using a flexible, non‐stretchable tape. Measurements were taken with the patient standing.

#### 2.3.2 Body composition analysis

Dual‐energy x‐ray absorptiometry (DXA, iDXA, General Electric Healthcare) was used to assess total fat mass, fat‐free mass (lean mass and bone mineral content), and visceral fat mass, expressed in kilograms and as a percentage of total body weight. Appendicular skeletal muscle mass (ASMM) was evaluated to diagnose sarcopenia based on standardised cut‐offs: ASMM/body weight <25.7% for men and <19.4% for women.^13^

#### 2.3.3 Energy expenditure

Resting energy expenditure (REE) was measured by indirect calorimetry (Quark RMR, Cosmed, Rome, Italy) for 30 min after a fasting period of 12 h. A calibration with a gas of known and certified CO_2_ and O_2_ composition was completed before starting the assessment (5% CO_2_, 16% O_2_, balanced with nitrogen). Measurements were standardised by internal guidelines. Before the measurement and the evening before, subjects had not been physically active. The subjects were in a supine position and awake, with the head placed in a clear ventilated canopy. Oxygen consumption and carbon dioxide production were measured, and energy expenditure was calculated by the Weir formula.^15^

#### 2.3.4 Muscle function

Grip Strength was measured using a Jamar hydraulic dynamometer on the dominant hand. Three attempts were recorded, with the highest value used for analysis.

Sarcopenia was defined as reduced grip strength (<27 kg for men and <16 kg for women) coupled with reduced ASMM.^16^ Dynapenia was defined solely as reduced grip strength.

#### 2.3.5 Bioelectrical impedance analysis (BIA)

Total water and third space water contents were assessed in the supine position using a multi‐frequency BIA, Bodystat Quadscan 4000, according to the manufacturer's recommendations. BIA resistance and reactance were measured at 50 kHz, while BIA impedance was determined at each frequency (5, 50, 100, and 200 kHz).

## 3 STATISTICAL ANALYSIS

No a priori sample size calculation was performed, as all consecutive eligible patients were included within the ATU and early access programme.

Data were analysed using GraphPad software (version 8.3.0). Normality was previously assessed by both Shapiro–Wilk and Kolmogorov–Smirnov tests. For the follow‐up at 0, 7, and 12 months, values were compared using non‐parametric tests for repeated measures (Friedman tests) with Dunn's multiple post hoc tests. For the comparison of change at 7 and 12 months, results were compared by using non‐parametric tests for paired data (Wilcoxon tests). To compare the clinical characteristics and the changes according to patient subgroups, values were analysed by using non‐parametric Mann–Whitney tests. Finally, Fisher's exact test was used to compare the sex distribution in patient subgroups and to evaluate the evolution of sarcopenic status. In all cases, a *p*‐value <0.05 was considered as significant.

## 4 RESULTS

### 4.1 Study participants

Between February 2022 and June 2023, 115 patients were enrolled and began treatment with Semaglutide. By the end of the seventh month, 9 patients had discontinued treatment and were excluded from the study. Among these, 7 stopped due to gastrointestinal side effects, 1 stopped due to gallstone‐related cholecystitis, and 1 due to worsening renal function in the context of pre‐existing chronic kidney disease.

A total of 106 patients were included in the study. Demographics and baseline clinical characteristics of patients are shown in Table 1. Most participants were female (68.9%), with a mean age of 52 years. The mean body weight was 127.2 kg, the mean BMI 46.3 kg/m^2^, and the mean waist circumference 130.9 cm; 91.5% had liver steatosis and 35.8% had type 2 diabetes. A history of bariatric surgery was reported in 21.7% of the patients. Among the 23 patients with a history of bariatric surgery, 19 had previously undergone sleeve gastrectomy, while the remaining had other procedures. The baseline characteristics of the study cohort, all of whom underwent DXA assessments, are provided in Table 1. 49% of patients exhibited sarcopenia at baseline.

**TABLE 1:** Demographic and clinical characteristics of the patients at baseline.

Characteristic	mean ± SD or *n* (%)
Age (year)	52.2 ± 12.1
Female sex—*n* (%)	73 (68.9)
Body weight—BW (kg)	127.2 ± 23.3
Height (m)	1.65 ± 0.09
BMI (kg.m^−2^)	46.3 ± 6.9
Waist circumference (cm)	130.9 ± 14.3
Handgrip strength (kg)	27.6 ± 10.8
Fat mass (kg)	65.5 ± 13.6
Fat mass (%)	51.8 ± 4.8
Visceral fat mass (kg)	3.10 ± 1.47
Lean mass (kg)	58.2 ± 12.1
Appendicular skeletal muscle mass (kg)	9.65 ± 1.56
Resting energy expenditure (kcal/day)	2149 ± 512
Hypertension—*n* (%)	69 (65.1)
Obstructive sleep apnea—*n* (%)	80 (75.5)
Liver steatosis—*n* (%)	97 (91.5)
Dyslipidemia—*n* (%)	50 (47.1)
Type 2 diabetes—*n* (%)	38 (35.8)
Sarcopenia—*n* (%)	52 (49.0)
Bariatric surgery—*n* (%)	23 (21.7)
Treatment with GLP‐1 analogs—*n* (%)	6 (5.7)

### 4.2 Changes in body weight

**FIGURE 1:**
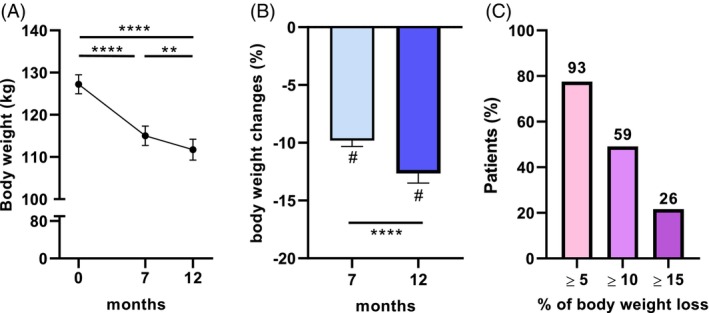
Effects of Semaglutide treatment on body weight. Effects of Semaglutide treatment over 12 months on body weight (A), change in body weight (B) and percentage of patients who met weight loss goals (C). Values are means ± sem in panels (A) and (B). In panel (C), the values above the bars indicate the number of patients. **, *p* <0.01; ****, *p* <0.0001.

Weight loss was significant during the first 7 months and continued up to 12 months, as shown in Figure 1A. Mean body weight loss was 9.8% at M7 and 12.7% at M12 (Figure 1B). 59% and 26% of the patients lost, respectively, at least 10% and 15% of body weight at the end of the study (Figure 1C).

### 4.3 Changes in body composition

**FIGURE 2:**
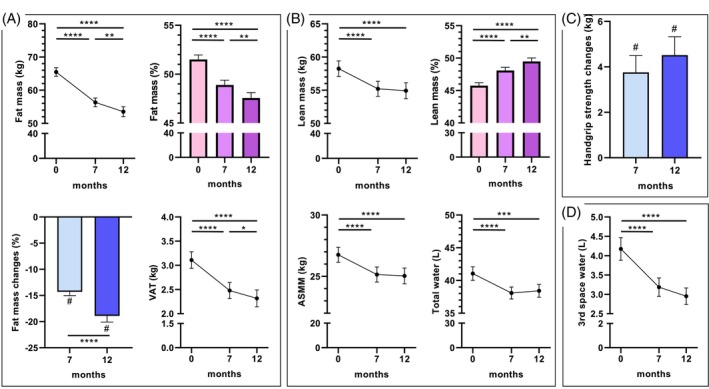
Change in Body composition induced by Semaglutide treatment. Effects of Semaglutide treatment over 12 months on body composition. Panel (A) shows the effects of Semaglutide on fat mass in kg, in % and on visceral adipose tissue (VAT) after 7 and 12 months and the percentage of fat mass loss. Panel (B) shows the effects of Semaglutide on lean mass in kg, in % and on appendicular skeletal muscle mass (ASMM) in kg and total water in Litre (L) after 7 and 12 months and the percentage of fat mass loss. Panel (C) shows the change in handgrip strength at 7 and 12 months, and Panel (D) shows the change in third space water in L. In all panels, values are means ± sem. *, *p* <0.05; **, *p* <0.01; ***, *p* <0.001; ****, *p* <0.0001.

Total fat mass decreased significantly at M7 (−14.3%) and continued up to M12 (−18.9%) as shown in Figure 2A, as well as visceral adipose tissue (VAT). Lean mass decreased significantly from baseline to M7, in absolute terms (−3.0 kg), but stabilised thereafter until M12 (Figure 2B). ASMM and total water exhibited the same evolution. The proportion of lean mass relative to total body mass increased significantly with Semaglutide, both at M7 and M12 compared to baseline. The third space water decreased significantly at M7 and M12 (Figure 2D).

### 4.4 Changes in muscle function and sarcopenia

Handgrip strength increased significantly both at M7 (+3.7 kg) and M12 (+4.1 kg) compared to baseline (Figure 2C). Patients with sarcopenic obesity decreased significantly from baseline (49%) to M12 (33%) (Figure 5). 22% of patients who were sarcopenic at baseline were no longer sarcopenic at M12; 49% of patients maintained their non‐sarcopenic status at M12; and 5% of patients developed sarcopenic obesity at M12 (Figure 5).

### 4.5 Changes in REE

**FIGURE 3:**
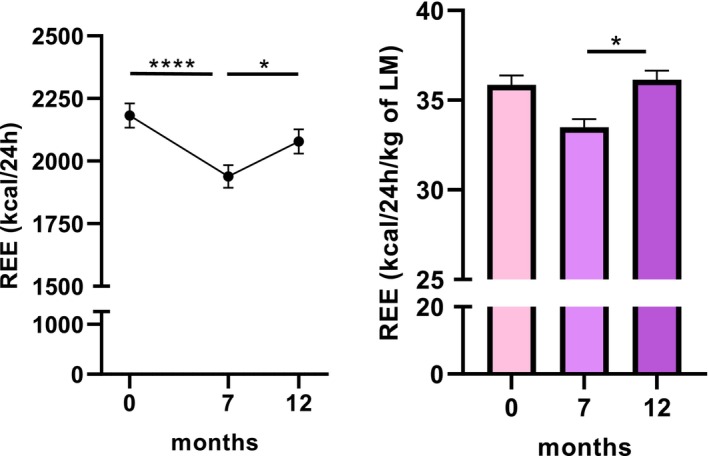
Effects of Semaglutide treatment on resting energy expenditure. Change in resting energy expenditure (REE) expressed in kcal/24 h (A) or normalised on lean mass (LM) (B) over 12 months of Semaglutide treatment. Values are means ± sem. *, *p* <0.05; ****, *p* <0.0001.

REE decreased significantly at M7 (−244 Kcal/24 h) compared to baseline, and then increased significantly at M12 (+140 Kcal/24 h) compared to M7 (Figure 3).

REE normalised to lean mass increased significantly from M7 to M12 (33.48 ± 4.50 Kcal/kg/24 h vs. 36.14 ± 5.13 Kcal/kg/24 h, *p* <0.05).

### 4.6 Impact of the sex, the diabetic comorbidity, previous treatments with GLP‐1 analogs, and bariatric surgery

**FIGURE 4:**
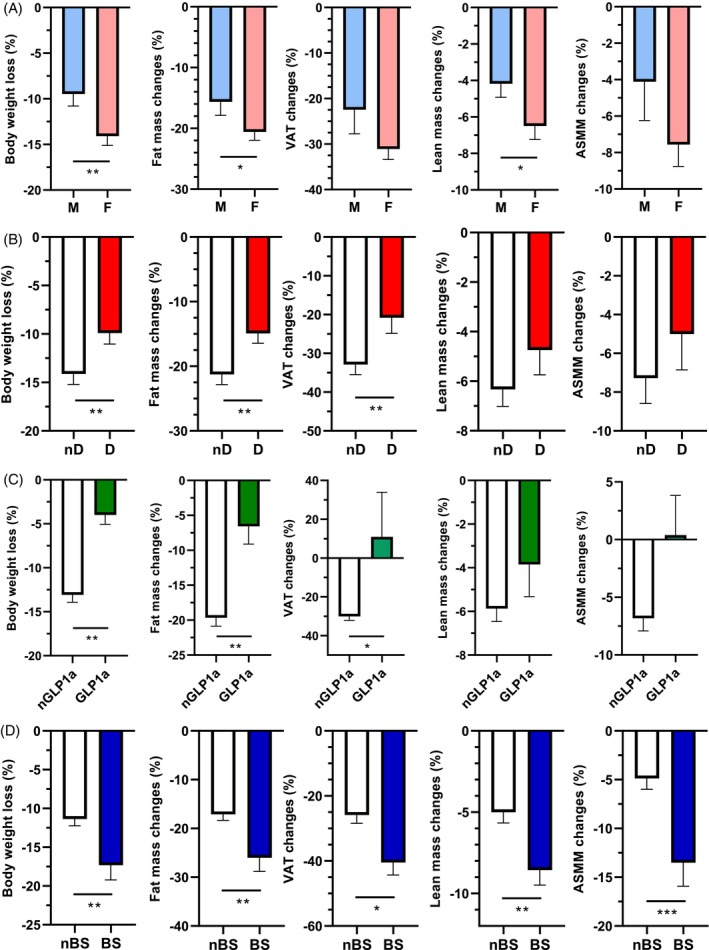
Effects of Semaglutide treatment on body weight loss and change in body composition according to the sex, the diabetic comorbidity, and previous treatments with GLP‐1 analogs or bariatric surgery. Effects of Semaglutide treatment during 12 months on the percentage of loss in body weight, fat mass, visceral adipose tissue (VAT), lean mass, and appendicular skeletal muscle mass (ASMM) in patients with obesity according to the sex (Panel A), to the presence (D) or not (nD) of type 2 diabetes diagnosis (Panel B), to previous treatments with GLP‐1 analogs (GLP‐1ra) or not (nGLP‐1ra) (Panel C), and to a history of bariatric surgery (BS) or not (nBS) (Panel D). In all panels, values are means ± sem. *, *p* <0.05; **, *p* <0.01; ***, *p* <0.001.

Decrease of body weight, fat mass, and lean mass was significantly higher in women than in men (Figure 4A). Patients with type 2 diabetes exhibited significantly lower body weight, fat mass, and VAT loss (Figure 4B). Previous treatments with GLP‐1 analogs were significantly associated with lower body weight and fat mass loss, and with a significant increase in VAT (Figure 4C). Patients with a history of bariatric surgery had significantly higher body weight, fat mass, lean mass, VAT, and ASMM loss (Figure 4D).

**FIGURE 5:**
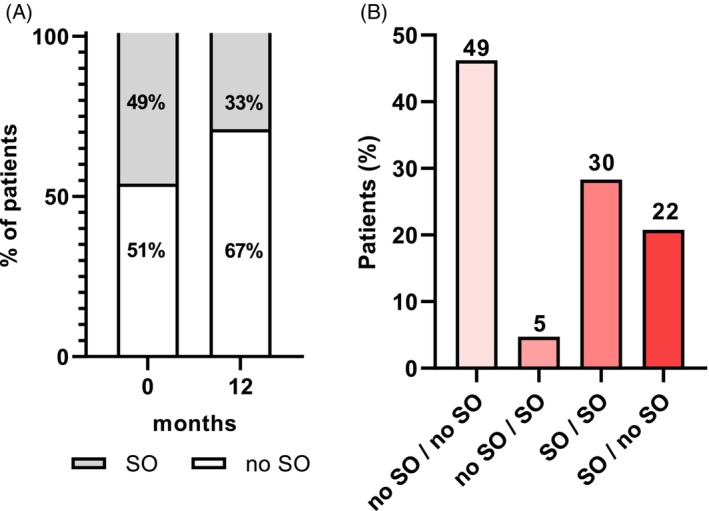
Percentage of patients with sarcopenic obesity before and after Semaglutide treatment. Panel (A) shows the percentage of patients with sarcopenic obesity that was calculated before and after 12 months of Semaglutide treatment. Open bars represent the patients with obesity without sarcopenia (no SO) and grey bars represent patients with sarcopenic obesity (SO). Panel (B) shows the percentage of patients according to the evolution of sarcopenic status (no SO vs. SO) before and after Semaglutide treatment. The values above the bars indicate the number of patients.

## 5 DISCUSSION

The SEMALEAN study shows the significant impact of the highest dose of Semaglutide (2.4 mg) on body weight loss in patients with obesity. Weight loss was significant during the first 7 months and continued through 12 months, with 59% of patients achieving a ≥ 10% weight loss and 26% achieving a ≥ 15% reduction. These findings are consistent with previous reports highlighting the efficacy of Semaglutide in promoting substantial weight loss.^6^ However, our study further advances existing knowledge by providing a longitudinal assessment of changes in fat mass, lean mass, muscle function, and metabolic adaptations over 12 months, using gold‐standard methods. This extended follow‐up offers new insights into the dynamics of body composition and functional outcomes during Semaglutide treatment.

Semaglutide treatment was associated with a significant decrease in fat mass and VAT at M7, which continued to decrease until M12. However, lean mass showed a different trajectory, with an initial significant decrease at M7, followed by stabilisation through M12. Interestingly, the proportion of lean mass relative to total body mass increased between M7 and M12, as well as the handgrip strength. Sarcopenic obesity decreased from 49% at baseline to 33% at M12. The preservation of muscle mass during Semaglutide treatment has also been reported by Wilding et al. in 140 patients with obesity who received the highest dose of Semaglutide (2.4 mg).^6^ Using DXA, an overall decrease in total lean mass (−9.7%) from baseline has been observed, but the proportion relative to total body mass increased by 3.0% points. Blundell et al. assessed the impact of 1 mg of Semaglutide in 30 patients with obesity over 12 weeks and reported a three‐fold greater loss of fat mass over lean mass using air displacement plethysmography.^11^ With the same Semaglutide dose over 24 weeks, in a population of 53 Chinese adults with obesity, Xiang et al. reported a significant weight loss, and muscle mass and muscle strength maintenance, measured respectively with BIA and the handgrip test.^9^ McCrimmon et al. evaluated the effects of Semaglutide 1.0 mg and canagliflozin 300 mg on body composition using DXA in 178 patients with type 2 diabetes over 52 weeks.^8^ Mean baseline BMI across all groups was 32 kg/m^2^. Although total lean mass was reduced with Semaglutide and canagliflozin, the proportion of lean mass increased by 1.2% and 1.1% point, respectively. In 48 patients with type 2 diabetes and a mean BMI of 36.9 kg/m^2^, Volpe et al. reported the effects of Semaglutide 1.0 mg over 52 weeks on body composition (BIA) and muscle function (handgrip). The ratio of skeletal muscle mass/VAT progressively increased, reaching significantly higher values than at baseline after 1 year of therapy, while handgrip strength was maintained during the study.^10^ In our study, ASMM decreased significantly, which is an important finding in the context of sarcopenia. However, this quantitative reduction was associated with a relative increase in lean mass proportion and an improvement in handgrip strength, suggesting that functional outcomes were maintained. These results emphasise the need to carefully monitor muscle mass and function during Semaglutide treatment, as loss of ASMM could predispose vulnerable patients to sarcopenia despite apparent improvements in body composition and strength.

All these data suggest lean mass and muscle function preservation during Semaglutide treatment. However, the involved mechanisms are still poorly studied. Few preclinical data reported beneficial effects of Semaglutide in obese mice. Ren et al. observed that obese high fat diet (HFD) mice exhibited a significant reduction of intramuscular fat, higher muscle protein synthesis, an increased relative proportion of skeletal muscle, and improved muscle function, possibly by altering the metabolism of muscle lipids.^17^ Xiang et al. also reported recently that Semaglutide protects skeletal muscle against obesity‐induced muscle atrophy in obese HFD mice, via the SIRT1 pathway.^18^ Further studies are needed to better understand these mechanisms. Interestingly, previous studies highlighted the positive effects of GLP‐1 receptor agonists on muscle protein synthesis and grip strength in mouse or rat models of cachexia or muscle atrophy.^19^, ^20^

Although our results suggest relative preservation of lean mass proportion and improvement in handgrip strength, recent reports have raised concerns regarding the potential adverse impact of Semaglutide on muscle mass and function, particularly in older adults with type 2 diabetes.^21^ These findings underscore the possibility of dose‐related effects and differential responses according to age or baseline muscle status. Taken together, these data highlight the need for careful monitoring of muscle health and functional capacity during Semaglutide treatment, especially in vulnerable populations at risk of sarcopenia.

In our study, we also reported that REE decreased at M7, reflecting the expected metabolic adaptation to weight loss,^22^ but increased significantly at M12 when normalised to lean mass. Clinically, the impact of Semaglutide on REE has been poorly studied. Blundell et al. reported no effect of once weekly Semaglutide 1.0 mg on REE adjusted for lean body mass.^11^ In mice, Gabery et al. have shown that Semaglutide reduces body weight through direct effects in the hypothalamus, as well as secondary effects in several areas involved in energy metabolism.^23^ By the end of the 11 day period, EE decreased in control mice that were weight‐matched to the Semaglutide group through food restriction, whereas EE in the Semaglutide‐treated group returned to baseline levels. This suggests that Semaglutide maintains metabolic activity despite weight loss, thereby aiding in prolonged weight loss or maintenance. Interestingly, recent data indicate that weight loss with Semaglutide treatment increases skeletal muscle mitochondrial efficiency in mice.^24^ Further studies are needed to elucidate the precise mechanisms behind these metabolic changes.

In our study, subgroup analyses revealed significant differences based on sex. Women experienced greater reductions in body weight, fat mass, and lean mass than men, which may reflect sex‐specific metabolic responses to Semaglutide. These results are in accordance with recent data in patients with obesity‐related HFpEF (heart failure with preserved ejection fraction) receiving Semaglutide 2.4 mg and exhibiting higher reduced body weight in women.^25^ Further data are needed to better understand the sex‐specific response to Semaglutide therapy. As reported in a recent review,^26^ several mechanisms may explain this difference: a higher proportion of subcutaneous adipose tissue, which is more responsive to weight loss; sex hormones such as oestrogens modulate appetite regulation, fat oxidation, and muscle metabolism; and women may also derive stronger satiety and appetite‐suppressing effects from GLP‐1 RAs compared with men. These findings suggest that biological sex is an important determinant of treatment response and should be considered in future personalised approaches.

Conversely, patients with type 2 diabetes exhibited smaller reductions in body weight, VAT, and fat mass, consistent with the known metabolic challenges in this population, including insulin resistance.^27^ However, in this population, Semaglutide also improves cardiometabolic risk factors and glycaemic control.^28^ Of note, the subgroup of patients previously treated with GLP‐1 receptor agonists was very small (*n* = 6), which limits the reliability of the findings and explains the wide variability observed. Baseline characteristics of this subgroup were comparable to those of unexposed patients (Supplemental Table S2), but these exploratory results should be interpreted with caution. In our study, patients with a history of bariatric surgery experienced greater losses of both fat and lean mass during Semaglutide treatment. Most of these patients (19 out of 23) had undergone sleeve gastrectomy, a restrictive procedure, suggesting that malabsorption is unlikely to be the main explanation. Rather, long‐term adaptations in gut hormone secretion (GLP‐1, PYY, ghrelin) may interact with the pharmacological effects of Semaglutide and potentiate weight loss.^29^ The greater lean mass loss in this subgroup also emphasises the importance of monitoring muscle and nutritional status in patients with a history of bariatric surgery, while data suggest that Semaglutide may complement surgical interventions, particularly in patients experiencing weight regain.^30^

The SEMALEAN's strengths include its comprehensive assessment of body composition, the assessment of muscle function, and the longitudinal evaluation of REE. Body composition was assessed using DXA, which is the gold standard for this purpose. Although BIA measurements were available, we did not use them in the analysis since BIA‐derived equations are known to be less accurate and not reliable in individuals with obesity, as previously reported.^31^ However, several limitations must be acknowledged. The relatively small sample size in certain subgroups and the exclusion of patients who discontinued treatment may limit the generalisability of our findings. Subgroup analyses (e.g., according to sex, diabetes status, prior GLP‐1 exposure, bariatric surgery) were exploratory and not adjusted for multiple comparisons. Additionally, the follow‐up was restricted to 12 months, precluding conclusions about long‐term outcomes beyond this period. Another limitation of our study is the absence of a control group. This reflects the real‐world design within the framework of the French temporary use authorisation and early access programme for Semaglutide, which did not allow for the inclusion of a comparator arm. As such, causal inference should be made with caution.

In conclusion, the SEMALEAN study highlights the impact of Semaglutide on obesity management, addressing not only weight loss but also lean mass, muscle function, and metabolic efficiency. These findings emphasise the importance of a comprehensive approach to obesity treatment that goes beyond weight loss alone.

## AUTHOR CONTRIBUTIONS

**MA, VF, NA**: Design; **MA, TD, VF, HVE, HL, JB, SGu, AlA, AgA, CR, AP, MG, PEC, LV, SGr, PD, GC, NA**: Data collection; **MA, TD, MC**: Analysis; **MA, MC, NA**: Writing—original draft. All authors: Writing—review and editing.

## FUNDING INFORMATION

No funding source to declare.

## CONFLICT OF INTEREST STATEMENT

Vanessa Folope is currently employed by Novo Nordisk. However, her involvement in the study design occurred before joining the company. The other authors declare no conflicts of interest related to this publication.

## Supporting information


Data S1

## Data Availability

The data that support the findings of this study are available from the corresponding author (NA), upon reasonable request.
