# Supplementary material for: Impact of Semaglutide on fat mass, lean mass and muscle function in patients with obesity: The SEMALEAN study
Source: Diabetes Obes Metab. 2025 Oct 9;28(1):112–21. doi: 10.1111/dom.70141 (PMC12673431; doi:10.1111/dom.70141)
Supplement: Supplementary file 1 — Data S1 [file DOM-28-112-s001.docx]

**Supplemental Table S1: Changes of body weight and composition after 12 months of GLP1a treatment**

|  | **All**  **(n=106)** | **Females**  **(n=73)** | **Males**  **(n=33)** |
| --- | --- | --- | --- |
| Body weight (kg) | -15.6 ± 10.6 | -16.6 ± 10.6 | -13.4 ± 10.4 |
| Body weight (%) | -12.6 ± 8.6 | -14.1 ± 8.7 | -9.5 ± 7.5 |
| Fat mass (kg) | -12.1 ± 8.0 | -12.9 ± 7.7 | -10.3 ± 8.5 |
| Fat mass (%) | -18.9 ± 12.3 | -20.6 ± 11.8 | -15.6 ± 12.9 |
| Visceral fat mass (kg) | -0.78 ± 0.63 | -0.59 ± 0.86 | -0.86 ± 0.94 |
| Lean mass (kg) | -3.27 ± 3.30 | -3.39 ± 3.4 | -3.00 ± 3.11 |
| Appendicular skeletal muscle mass (kg) | -1.73 ± 3.06 | -2.14 ± 3.54 | -1.53 ± 4.23 |

Values are means ± standard deviation.

**Supplemental Table S2: Demographic and clinical characteristics of patients at baseline according to their subgroups**

|  | **Females**  **(n=73)** | **Males**  **(n=33)** | **nD**  **(n=68)** | **D**  **(n=38)** | **nGLP1a**  **(n=100)** | **GLP1a**  **(n=6)** | **nBS**  **(n=83)** | **BS**  **(n=23)** |
| --- | --- | --- | --- | --- | --- | --- | --- | --- |
| Age (y) | 53.2 ± 10.2 | 50.2 ± 15.7 | 49.5 ± 11.2 | 56.9 ± 12.4**^b^** | 52.4 ± 12.1 | 59.6 ± 11.1 | 51.8 ± 13.2 | 53.3 ± 7.4 |
| Female sex – n (%) | - | - | 49 (72) | 24 (63) | 69 (69) | 4 (66.6) | 54 (65) | 19 (82.6) |
| Body weight - BW (kg) | 119.1 ± 7.0 | 145.5 ± 21.6**^a^** | 128.4 ± 24.2 | 125.2 ± 22.2 | 127.5 ± 23.9 | 122.4 ± 8.5 | 128.1 ± 23.1 | 124.1 ± 25.0 |
| Height (m) | 1.61 ± 0.07 | 1.75 ± 0.07 | 1.66 ± 0.09 | 1.64 ± 0.09 | 1.65 ± 0.10 | 1.62 ± 0.08 | 1.66 ± 0.10 | 1.62 ± 0.09 |
| BMI (kg.m^-2^) | 45.8 ± 6.7 | 47.4 ± 7.3 | 46.4 ± 7.0 | 46.2 ± 6.8 | 46.4 ± 7.0 | 45.9 ± 4.3 | 46.1 ± 6.7 | 47.1 ± 7.7 |
| Waist circumference (cm) | 126.5 ± 13.3 | 141.7 ± 10.4 **^a^** | 128.9 ± 13.3 | 134.6 ± 15.8 | 130.7 ± 14.8 | 133.5 ± 4.4 | 133.2 ± 13.8 | 124.9 ± 14.6**^c^** |
| Handgrip Strength (kg) | 22.3 ± 5.9 | 39.2 ± 9.8 **^a^** | 29.4 ± 10.6 | 24.2 ± 10.5 **^b^** | 27.9 ± 10.8 | 19.2 ± 6.7 | 28.4 ± 11.2 | 23.3 ± 7.4 |
| Fat mass (kg) | 63.6 ± 13.1 | 69.7 ± 13.9 **^a^** | 66.9 ± 14.0 | 63.2 ± 12.8 | 65.6 ± 13.9 | 63.4 ± 4.1 | 65.3 ± 13.2 | 66.6 ± 15.2 |
| Fat mass (%) | 53.5 ± 3.9 | 47.8 ± 4.4 **^a^** | 52.4 ± 4.8 | 50.9 ± 4.8 | 51.9 ± 4.9 | 51.5 ± 3.1 | 51.3 ± 4.9 | 53.9 ± 4.1 **^c^** |
| Visceral fat mass (kg) | 2.51 ± 0.99 | 4.42 ± 1.53 **^a^** | 2.80 ± 1.20 | 3.60 ± 1.76 | 3.10 ± 1.49 | 3.10 ± 1.07 | 3.30 ± 1.52 | 2.17 ± 0.75**^c^** |
| Lean mass (kg) | 52.1 ± 7.2 | 71.7 ± 9.8 **^a^** | 58.0 ± 12.9 | 58.2 ± 11.0 | 58.1 ± 12.4 | 57.3 ± 5.8 | 59.1 ± 12.1 | 54.5 ± 12.0 |
| Appendicular skeletal muscle mass (kg) | 23.6 ± 3.6 | 33.7 ± 5.1 **^a^** | 26.9 ± 6.6 | 26.4 ± 5.8 | 26.8 ± 6.4 | 24.5 ± 3.6 | 27.2 ± 6.4 | 24.8 ± 5.4 |

Values are means ± standard deviation. Data from Patients with obesity according to the sex, to the presence (D) or not (nD) of type 2 diabetes diagnose, to previous treatments with GLP1 analogs (GLP1a) or not (nGLP1a) and to an history of bariatric surgery (BS) or not (nBS). **^a^** p<0.05 vs females; ^b^ p<0.05 vs nD; ^c^ p<0.05 vs nBS (Mann-Whitney test).
